# Atypical Femoral Fracture in Hypophosphatasia: A Systematic Review

**DOI:** 10.1155/2023/5544148

**Published:** 2023-09-12

**Authors:** Nipith Charoenngam, Jerapas Thongpiya, Pitchaporn Yingchoncharoen, Ben Ponvilawan, Mehmet S. Marangoz, Jirat Chenbhanich, Patompong Ungprasert

**Affiliations:** ^1^Department of Medicine, Mount Auburn Hospital, Harvard Medical School, Cambridge, MA, USA; ^2^Department of Medicine, Faculty of Medicine Siriraj Hospital, Mahidol University, Bangkok, Thailand; ^3^Department of Medicine, Texas Tech University Health Sciences Center, Lubbock, TX, USA; ^4^Department of Medicine, University of Kansas Missouri Medical Center, Jefferson, MO, USA; ^5^Department of Endocrinology and Metabolism, Mount Auburn Hospital, Cambridge, MA, USA; ^6^Department of Genetics and Genomic Sciences, Case Western Reserve University, Cleveland, Ohio, USA; ^7^Department of Rheumatic and Immunologic Diseases, Cleveland Clinic, Cleveland, Ohio, USA

## Abstract

**Objective:**

To summarize the characteristics of all reported patients with hypophosphatasia (HPP) who sustained atypical femoral fracture (AFF) and identify all available evidence to quantify the rate of coexistence between HPP and AFF.

**Methods:**

Potentially eligible articles were identified from the MEDLINE and EMBASE databases from its inception to September 2022, using a search strategy consisting of terms related to “Hypophosphatasia” and “Atypical femoral fracture.” Eligible articles must report one of the following information: (1) individual data of patients diagnosed with HPP and AFF, (2) prevalence of HPP among patients with AFF, or (3) prevalence of AFF among patients of HPP. Characteristics of patients reported in each study were extracted.

**Results:**

A total of 148 articles were identified. After the systematic review, 24 articles met the eligibility criteria. A total of 28 patients with AFF and HPP were identified. The mean ± SD age of the reported patients was 53.8 ± 12.5 years, and 22 patients (78.6%) were female. Nine patients (32.1%) received antiresorptive medication (bisphosphonate and/or denosumab), and two patients (7.1%) received teriparatide prior to the development of AFF. Seven (25.0%) and eighteen (64.3%) patients sustained unilateral and bilateral AFF, respectively (laterality not reported in three cases). Thirteen patients (46.4%) had a history of fractures at other sites. Four (14.3%) and seven (25.0%) patients received asfotase alfa and teriparatide after sustaining AFF. Two studies reported the prevalence of AFF among patients with HPP of approximately 10%. One study reported one HPP patient in a cohort of 72 patients with AFF.

**Conclusions:**

Based on the limited evidence, AFF occurred in up to 10% of patients with HPP. Based on the 28 case reports, about two-thirds did not receive antiresorptive treatment, suggesting that the HPP itself could potentially be a risk factor for AFF.

## 1. Introduction

Hypophosphatasia (HPP) is a hereditary metabolic bone disorder caused by loss-of-function pathogenic variants of the ALPL gene that encodes tissue-nonspecific-isoenzyme of alkaline phosphatase (TNSALP) [1]. TNSALP is a phosphohydrolase enzyme expressed in several organs including the bones, developing teeth, liver, and kidneys. In the skeletal and dental tissues, TNSALP converts inorganic pyrophosphate into inorganic phosphate, which is crucial for crystallization of calcium hydroxyapatite [1, 2]. Defective calcium hydroxyapatite formation in HPP results in demineralization of skeletal and dental tissues, which, in turn, leads to osteomalacia, skeletal fragility, and teeth loss. Other consequences of HPP include development of calcium pyrophosphate deposition due to accumulation of circulating inorganic pyrophosphate and pyridoxine-dependent seizure due to impaired *γ*-carboxyglutamic acid synthesis [3, 4].

HPP exists in several forms, depending on the severity of TNSALP deficiency. It can be categorized based on age of onset and severity, including odontoHPP, adult HPP, childhood HPP, infantile HPP, and perinatal HPP (ranked in increasing severity) [1, 3, 4]. Severe HPP is rare with the incidence of approximately 1 per 100,000 live births; however, milder forms of HPP can be more prevalent and underdiagnosed [5, 6].

Atypical femur fracture (AFF) is an uncommon type of subtrochanteric or femoral shaft fracture occurring with minimal trauma or no trauma and have specific radiographic findings [7]. This condition has emerged as potential complication of long-term use of antiresorptive osteoporosis therapy including bisphosphonates and denosumab or anabolic agents including romosozumab [8–11]. As a result, AFF has become a major concern for clinicians despite the proven efficacy of these medications for the prevention of major osteoporotic fracture and the very low incidence of AFF of only 1.8–113 cases per 100,000 person-year (age adjusted incidence rate). In addition, one-year mortality risk of AFF is relatively low with the reported incidence of approximately 10% [12, 13].

Given the unclear pathogenesis, it is still not known with certainty who is particularly susceptible to AFF. Long duration of bisphosphonate exposure, glucocorticoid use, and Asian race were found to be associated with an increased risk of AFF in an epidemiological study [8]. In addition, variant in the GGPS1 gene has been associated with bisphosphonate-related AFF based on whole-exome sequencing of three sisters [14]. Interestingly, a growing number of case reports and case series have demonstrated that AFF can occur in patients with HPP [15–36]. Due to the lack of summary of the clinical manifestation, risk factors, associated ALPL variants, and treatment, we conducted this systematic review with the goal to summarize the characteristics of all reported patients with HPP who sustained AFF. Moreover, we aimed at identifying all available evidence to quantify the rate of coexistence between HPP and AFF.

## 2. Method

### 2.1. Search Strategy

Three investigators (N.C., J.T., and B.P.) independently searched published studies indexed in MEDLINE and EMBASE databases from inception to September 2022. The search terms were derived from terms related to “Atypical femoral fracture” and “Hypophosphatasia.” The search strategy is shown in Supplementary Material 1. No language limitation was applied.

### 2.2. Eligibility Criteria

The eligible articles must report one of the following information: (1) individual data of a patient or patients diagnosed with HPP and AFF, (2) prevalence of HPP in a cohort of patients with AFF, or (3) prevalence of AFF in a cohort of patients of HPP. Reviews of published cases were excluded to avoid case duplication. Letters to the editor were included if they met all eligibility criteria.

Two investigators (N.C. and J.T.) independently reviewed each article to determine its eligibility. For articles that did not explicitly report AFF, ascertainment of AFF was determined based on case descriptions verified by the 2013 American Society for Bone and Mineral Research Case Definition of Atypical Femoral Fracture [37]. Different opinions regarding the eligibility of some reports were resolved by discussions with the senior author (P.U.).

### 2.3. Data Extraction

Standardized data collection forms were applied for data extraction. For studies reporting individual patient data, the following details were extracted: last name of the first author, publication year, country, and individual patient data, including age, sex, antiosteoporosis medication exposure prior to the development of AFF, unilateral/bilateral AFF, presence of other fracture(s), ALPL variants, and medical treatment after AFF. For studies reporting prevalence of AFF among HPP patients and prevalence of HPP among AFF patients, the following details were extracted: last name of the first author, publication year, country, recruitment of participants, diagnosis of AFF, diagnosis of HPP, mean age of the participants, percentage of female participants, and number of cases with AFF and/or HPP.

## 3. Results

### 3.1. Search Results

**Figure 1:**
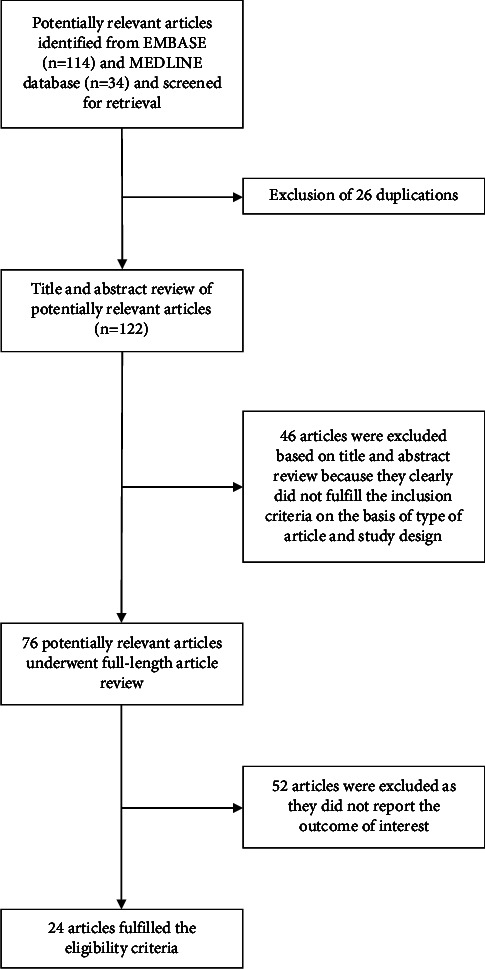
Study identification and literature review process.

A total of 148 articles were retrieved from EMBASE and MEDLINE databases. A total of 26 duplicated articles were withdrawn, leaving 122 articles for title and abstract review. After screening of the title and abstract, a total of 46 articles were excluded as they obviously did not meet the eligibility criteria based on the type of the article. This left 76 potentially eligible articles for full-length article review. A total of 52 articles were further excluded as they did not fulfill the eligibility criteria. Finally, 24 articles fulfilled the eligibility criteria [15–36, 38, 39].  Figure 1 demonstrates the search methodology and selection process of this study.

### 3.2. Summarizing the Evidence

Among the 24 included articles, 22 articles reported individual data of patients with HPP who developed AFF [15–36]. A total of 28 patients with AFF and HPP were identified from these 22 articles, most of which were reported in the United States and Europe, while few were from Australia, Canada, India, and Iran. The mean ± SD age of the reported patients was 53.8 ± 12.5 years, and 22 patients (78.6%) were female. Two patients (7.1%) had childhood HPP, and 26 patients (92.9%) had adult HPP. Nine patients (32.1%) received antiresorptive medication (bisphosphonate and/or denosumab), and two patients (7.1%) received teriparatide prior to the development of AFF. Seven (25.0%) and eighteen (64.3%) patients sustained unilateral and bilateral AFF, respectively (laterality not reported in three cases). Thirteen patients (46.4%) had a history of fractures at other sites. Single heterozygous, compound heterozygous, and homozygous ALPL variants were reported in six (21.4%), nine (32.1%), and two (7.1%) patients, respectively. Four (14.8%) and seven (25.0%) patients received asfotase alfa and teriparatide after sustaining AFF. The characteristics of the 28 included patients are summarized in Table 1.

There are two studies that reported the prevalence of AFF among patients with HPP. In a single center study by Genest and Seefried [38], 150 patients in Germany diagnosed with HPP by clinical signs/symptoms and biochemical and genetic tests were reviewed. Fifteen patients (mean age 55 years, 75% female) out of the 150 studied patients were found to have AFF based on review of medical record. Eight out of fifteen patients who developed AFF never received treatment with bisphosphonate or denosumab. In a study of 19 patients (mean age 45 years, 53% female) with HPP, who attended the Centre for Metabolic Bone Diseases and the Centre for Metabolic Diseases, University Hospitals Leuven, Belgium, two patients were found to have AFF [26]. Both received bisphosphonate or denosumab prior to the fracture. Taken together, the prevalence of AFF among patients with HPP based on the limited evidence was approximately 10% (almost half of them never received bisphosphonate or denosumab).

One study reported the frequency of HPP in a cohort of AFF. A retrospective study by Tsiantouli et al. [39] included 72 patients with confirmed AFF by chart review and found that 18 (25%) patients had at least one serum ALP concentration of ≤40 IU/L. Among these 18 patients with low ALP concentrations, one patient was confirmed to have HPP with a pathogenic heterozygous variant (c.787T > C, p.Tyr263His) in ALPL. However, no individual data of the patient were reported.

## 4. Discussion

The pathogenesis of AFF is still not well understood but is thought to start as a stress fracture on the lateral femoral cortex due to high-tensile loads, which is predisposed by multiple factors such as increased mineral content, reduced heterogeneity of tissue properties, and accumulation of microdamage [40, 41]. Antiresorptive osteoporosis medications including bisphosphonates and denosumab are believed to inhibit stress fracture healing, thereby impairing the ability of the skeletal tissue to resist overt fracture [40, 41]. To date, long-term exposure to bisphosphonates or denosumab is the only well-established risk factor AFF.

This study is the first systematic review that comprehensively investigated the coexistence of AFF and HPP. We identified a total of 28 reported cases of AFF in patients with HPP with variable disease severity and genetic variants in the ALPL gene. Based on the limited evidence (two cohort studies), AFF occurred in up to 10% of patients with HPP and about half of them never received treatment with bisphosphonate or denosumab. In addition, based on the 28 case reports, about two-thirds did not receive antiresorptive treatment, suggesting that the HPP itself could potentially be a risk factor for AFF. Further studies with more vigorous study design are still required.

Studies have attempted to identify the genetic risk of AFF. Although no common genetic polymorphism was found to be associated with AFF in a genome-wide association study [42], several rare pathogenic variants have been associated with AFF. Most of them cause known monogenic disorders, such as osteogenesis imperfecta (*COL1A1/COL1A2*), pycnodysostosis (*CTSK*), X-linked osteoporosis (*PLS3*), osteopetrosis, X-linked hypophosphatemia (*PHEX*), and osteoporosis pseudoglioma syndrome (*LRP5*) [43]. Furthermore, by utilizing whole-exome sequencing, the p.Asp188Tyr variant in the *GGPS1* gene, a site of inhibition by bisphosphonates in the mevalonate pathway, was identified in three sisters with AFF associated with bisphosphonate exposure [14]. The pathogenicity of the variant was later supported by a functional study [44]. The aggregated data from our systematic review may support HPP as a predisposing condition of AFF, and one could hypothesize that variations in the ALPL gene may be another genetic predisposing factor of AFF.

Considering HPP a highly spectral disease, it can be speculated that low serum alkaline phosphatase (ALP) may be a risk factor for AFF associated with antiresorptive medication exposure. However, in a small case-control study of 10 AFF patients with 13 controls, the rate of low serum ALP (<55 U/L) was not significantly different between the two groups (5/10 in AFF groups and 5/13 in controls). Further investigations are needed to determine if low serum ALP is a marker of risk of AFF associated with antiresorptive medications [45].

Data on efficacy and safety of antiresorptive medications in HPP are very limited as patients with HPP are excluded from clinical trials [46–48]. In theory, the use of bisphosphonates could have a detrimental effect on patients with HPP because they are pyrophosphate analogs, which could further suppress the TNSALP activity and bone remodeling [3]. The observation that patients with HPP appeared to have a higher frequency of AFF and almost half of them in the cohort studies were exposed to antiresorptive medications before the onset of AFF may even raise more concern over their safety profile in this group of patients.

This systematic review carries some limitations that should be acknowledged. First, case reports and case series provide the lowest level of evidence. Thus, the strength of causal association between HPP (with and without antiresorptive exposure) and AFF cannot be determined. Second, the diagnostic accuracy of HPP in many case reports was limited due to incomplete data on genetic analysis. Furthermore, although patients in our cohort clinically fit with HPP, most of the studies did not report the variant classification according to the American College of Medical Genetics and Genomics. For example, the variant from the study by Righetti et al. [30] is of uncertain significance at present according to the ClinVar database (https://www.ncbi.nlm.nih.gov/clinvar/). Finally, the absence of data on the long-term outcomes of patients treated with asfotase alfa and teriparatide after AFF diagnosis hinders an evaluation of the efficacy of the treatments. Further studies are still needed to determine the therapeutic strategies of AFF in patients with HPP.

## 5. Conclusion

Based on the limited evidence, AFF occurred in up to 10% of patients with HPP. Based on the 28 case reports available in the literature, about two-thirds did not receive antiresorptive treatment, suggesting that the HPP itself could potentially be a risk factor for AFF. Further studies with more vigorous study design are still required.

## Figures and Tables

**Table 1:** Characteristics of reported patients with hypophosphatasia and atypical femoral fracture.

Patient no	Study	Year	Country	Age, sex	Type of HPP	Antiosteoporosis medication exposure	Unilateral/Bilateral	Other fracture(s)	*ALPL* variants	Medical treatment after AFF
1	Anaraki et al. [15]	2018	US	76F	adultHPP	AL (4 years), TPT (1 year), AL&ZA (3 years), ZA (3 years), DMAB (1 dose), TPT (1 year), followed by DMAB	Unilateral	Right wrist, right metatarsal, right knee cap, left metatarsal and lumbar spine fractures	N/A	—
2	Bhadada et al. [16]	2020	India	47F	adultHPP	—	Bilateral	—	Heterozygous: c.311A > G (p.Asn104Ser)	—
3	Buklemishev and Rodionova [17]	2020	Russia	40M	childhoodHPP	—	Bilateral	—	Compound heterozygous: c.182G > A (p.Gly61Glu), c.571G > A (p.Glu191lys)	AA and alfacalcidol
4	Chittimala et al. [18]	2020	US	56M	adultHPP	—	Bilateral	—	N/A	—
5	Coe et al. [19]	1986	US	67F	adultHPP	—	Bilateral	—	N/A	—
6	Coe et al. [19]	1986	US	61F	adultHPP	—	Unilateral	—	N/A	—
7	Coe et al. [19]	1986	US	66F	adultHPP	—	Bilateral	—	N/A	—
8	Coe et al. [19]	1986	US	69F	adultHPP	—	Bilateral	—	N/A	—
9	Coe et al. [19]	1986	US	45M	adultHPP	—	Unilateral	—	N/A	—
10	Doshi et al. [20]	2009	US	53F	adultHPP	RIS	Bilateral	—	N/A	TPT
11	Fanous and Barb [21]	2020	US	49F	adultHPP	—	Unilateral	Multiple metatarsal stress fractures and a rib fracture	Heterozygous: c.407G > A (p.Arg136His)	AA
12	Gagnon et al. [22]	2010	Australia	53F	adultHPP	—	Bilateral	Wrist and thoracic vertebral fractures	Compound heterozygous: c.526G > A (p.Ala176Thr), c.1268T > C (Val423Ala)	TPT
13	Klidaras et al. [23]	2018	US	41F	adultHPP	—	Bilateral	Rib and left tibial fractures	Compound heterozygous: c.526G > A (p.Ala176Thr), c1132G > C (p.Asp378His)	AA
14	Laurent et al. [24]	2017	France	69F	childhoodHPP	RIS (6 y) followed by DMAB	Unilateral	Wrist, metatarsal and tibial fractures	Heterozygous: c.361G > A (p.Val121Met)	—
15	Lawrence et al. [25]	2017	UK	55F	adultHPP	—	Bilateral	Right tibia and 4^th^ and 5^th^ metatarsal fractures	Compound heterozygous: c.526G > A p.(Ala176Thr), c.1171C > T p.(Arg391Cys)	—
16	Lefever et al. [26]	2020	Belgium	36F	adultHPP	BP and/or DMAB	N/A	—	Homozygous: c.896T > C (p.Leu299Pro)	—
17	Lefever et al. [26]	2020	Belgium	69F	adultHPP	BP and/or DMAB	N/A	—	Homozygous: c.896T > C (p.Leu299Pro)	—
18	Maman et al. [27]	2016	France	51M	adultHPP	—	Bilateral	Multiple metatarsal fractures	Compound heterozygous: T (p.Thr100Met), c.571G > A (p.Glu191Lys)	—
19	Miller et al. [28]	2017	US	61M	adultHPP	TPT (6 m)	Bilateral	—	Compound heterozygous: A (p.Pro292Thr), c.1195G > A (p.Ala399Thr)	AA
20	Resch et al. [29]	2016	Austria	25M	adultHPP	—	N/A	—	N/A	—
21	Righetti et al. [30]	2017	France	67F	adultHPP	AL	Bilateral	Fibular, ankle, and metatarsal fractures	Heterozygous: c.341C > T (p.Ala114Val)	TPT
22	Schalin-Jantti et al. [31]	2010	France	56F	adultHPP	—	Bilateral	Metatarsal fractures	Compound heterozygous: c.1015G > A (p.Gly339Arg), c.571G > A (p.Glu191Lys)	TPT
23	Schalin-Jantti et al. [31]	2010	France	64F	adultHPP	—	Unilateral	—	Compound heterozygous: c.1015G > A (p.Gly339Arg), c.571G > A (p.Glu191Lys)	TPT
24	Schini and Eastell [32]	2018	UK	48F	adultHPP	AL	Bilateral	2 metatarsal fractures	—	—
25	Siami et al. [33]	2022	Iran	30F	adultHPP	—	Bilateral	—	—	—
26	Sutton et al. [34]	2012	Canada	55F	adultHPP	AL followed by ZA	Bilateral	Left lower tibia stress periostosis, right 2^nd^ metatarsal, left 2^nd^ and 3^rd^ metatarsal, right distal radius, right distal ulna and right talus avulsion fractures	Heterozygous: A (p.Arg71His)	—
27	Warren et al. [35]	2021	Australia	40F	adultHPP	DMAB	Bilateral	Left navicular and 3^rd^ and 5^th^ metatarsal fractures	Compound heterozygous: A (p.Ala176Thr), c.881A > C (p.Asp294Ala)	TPT
28	Whyte et al. [36]	2007	US	56F	adultHPP	—	Unilateral	Right 4^th^ and 5^th^ metatarsal and left 5^th^ metatarsal stress fractures	Heterozygous: T (p.Asp378Val)	TPT

Abbreviations: AA: asfotase alfa; AL: alendronate; BP: bisphosphonate; DMAB: denosumab; F: female; M: male; N/A: not available; RIS: risedronate; TPT: teriparatide; UK: the United Kingdom; US: the United States; ZA: zoledronic acid.

## Data Availability

No data were used to support the findings of this study.

## Supplementary Materials

Supplementary Materials Supplemental material 1: searching strategy.
