# Supplementary material for: Atypical Femoral Fracture in Hypophosphatasia: A Systematic Review
Source: Int J Endocrinol. 2023 Sep 12;2023:5544148. doi: 10.1155/2023/5544148 (PMC10508997; doi:10.1155/2023/5544148)
Supplement: Supplementary Materials — Supplemental material 1: searching strategy. [file 5544148.f1.docx]

**Supplemental material 1** – Searching strategy

**EMBASE Database**

1. 'hypophosphatasia'/exp OR 'hypophosphatasia'
2. 'atypical femoral fracture'/exp OR 'atypical femoral fracture'
3. 'atypical fracture'/exp OR 'atypical fracture'
4. 'femur subtrochanteric fracture'/exp OR 'femur subtrochanteric fracture'
5. 'femur fracture'/exp OR 'femur fracture'
6. #2 OR #3 OR #4 OR #5
7. #1 AND #6

**Ovid MEDLINE Database**

1. hypophosphatasia.mp. or Hypophosphatasia/
2. atypical femoral fracture.mp.
3. atypical fracture.mp.
4. subtrochanteric fracture.mp.
5. femur fracture.mp.
6. femoral fracture.mp. or exp Femoral Fractures/
7. 2 or 3 or 4 or 5 or 6
8. 1 and 7
